# Is a patient-specific drill template via a cortical bone trajectory safe in cervical anterior transpedicular insertion?

**DOI:** 10.1186/s13018-018-0810-5

**Published:** 2018-04-18

**Authors:** Peng Peng, Yafei Xu, Xintao Zhang, Meisong Zhu, Bingran Du, Wenrui Li, Wenhua Huang, Jun Song, Jianyi Li

**Affiliations:** 1 Department of Anatomy, Guangdong Provincial Key Laboratory of Medical Biomechanics, School of Basic Medical Sciences, Southern Medical University, 1063 Shatai Nan Road, Baiyun District, Guangzhou, Guangdong China; 2 Department of Orthopedics, Nanhai Hospital, Southern Medical University, 28 Liguan Road, Nanhai District, Foshan, Guangdong China; 3 Department of Radiology, The Third Affiliated Hospital, Southern Medical University, 183 Zhongshandadao Xi, Tianhe District, Guangzhou, Guangdong China; 4 Department of Orthopedics, Zhujiang Hospital, Southern Medical University, 253 Gongye Street, Haizhu District, Guangzhou, Guangdong China; 5 General Education Department, Southern Medical University, 1063 Shatai Nan Road, Baiyun District, Guangzhou, Guangdong China

**Keywords:** Cortical bone trajectory, Patient-specific drill template, Computer numerical control, 3D printing, Computed tomography, Cervical anterior transpedicular insertion

## Abstract

**Background:**

This study aimed to develop patient-specific drill templates by computer numerical control or three-dimensional printing via two cortical bone trajectories (CBTs) and to evaluate their efficacies and accuracies in cervical anterior transpedicular insertion.

**Methods:**

Preoperative CT images of 20 cadaveric cervical vertebrae (C3–C7) were obtained. After image processing, patient-specific drill templates were randomly assigned to be constructed via two CBTs (CBT0 and CBT0.7) and manufactured by two methods (computer numerical control and three-dimensional printing). Guided by patient-specific drill templates, 3.5-mm-diameter screws were inserted into the pedicles. Postoperative CT scans were performed to evaluate the screw deviation in the entry point and midpoint of the pedicle. The screw positions were also graded.

**Results:**

Computer numerical control patient-specific drill templates had a significantly shorter manufacturing time compared to three-dimensional-printed patient-specific drill templates (*p* < 0.01). Absolute deviations at the entry point and midpoint of the pedicle had no significant differences on the transverse and sagittal planes (*p* > 0.05). There were no significant differences in screw positions (*p* = 0.3). However, three screw positions were in grade 3 in CBT0, while the others were in grade 1.

**Conclusions:**

CBT0.7 appears to be a safe and feasible trajectory for cervical anterior transpedicular insertion. Bio-safe computer numerical control patient-specific drill templates can facilitate cervical anterior transpedicular insertion with good feasibility and accuracy.

## Background

Anterior cervical decompression and fusion (ACDF) is widespread to treat the subaxial cervical spine diseases [1, 2]. However, the biomechanical stability is unsatisfied in the cases of single-level three-column injuries or multi-level anterior compression treated with anterior vertebral body screws [3, 4]. As an alternative, the anterior transpedicular screw (ATPS) technique was introduced by Aramomi et al. [5], and it has gradually been more widely applied to cervical stabilization [6–10] since it combines the advantages of an anterior approach with the superior biomechanical characteristics of cervical pedicle fixation [8]. Generally, pedicle fixation is considered risky because it is proximal to the vital structures such as the vertebral arteries, the spinal cord, and the nerve roots [11, 12]. Therefore, accurate anterior transpedicular insertion (ATPI) is a key to successful clinical application of ATPS.

Much research has been conducted to achieve accurate ATPI in the cervical spine with fewer risks. Koller et al. [9] reported successful ATPI using the fluoroscopic-guided freehand technique in cadaver research. However, the incidence of a critical pedicle breach in the axial plane was 21.7%. Computer-assisted navigation (CAN) systems were used in ATPI [13]. Patton et al. found that catastrophic screw placement occurred in 33.3% of cases performed by the fluoroscopic-guided freehand technique; that by CAN was significantly lower, but it was still 16.7%. Patient-specific drill templates (PDTs) produced by three-dimensional printing (3DP) were then introduced to assist with cervical transpedicular insertion with good effectiveness and accuracy [14, 15]. However, the materials used in 3DP are mainly photosensitive resin, which is not biocompatible and cannot be sterilized by high temperatures. Fu et al. [16] tried to develop a biocompatible PDT for ATPI using a bone cement mold according to a 3DP model of the cervical vertebrae with preset screw trajectories. However, their pedicle cortex penetration rate in critical positions was as high as 8.3%, which indicated that it was not an ideal solution for ATPI. Kong et al. [17] developed a bio-safe metal PDT using computer numerical control (CNC) and verified its high accuracy for posterior thoracic pedicle insertions. However, there have been no studies related to PDT by CNC in cervical applications.

Regarding the trajectory, the current PDTs usually use the center line (CL) of the pedicle as their ideal trajectory, where the screw is mainly engaged with cancellous bone in the pedicle and vertebral body [6, 8, 18, 19]. In this situation, it was reported that screw loosening might occur, leading to a loss of correction and nonunion, particularly in patients with poor bone quality [20, 21]. Therefore, Santoni et al. [22] advocated the cortical bone trajectory (CBT), which allowed the screw’s thread to contact the cortical bone. It was deemed an acceptable alternative to the CL trajectory for lumbar pedicle screw insertion. Biomechanical studies further demonstrated that the CBT technique achieves a screw purchase and strength greater than the traditional CL trajectory [22–24]. However, no study has been conducted using CBT in the cervical vertebrae.

Therefore, the aims of the study were to develop PDTs with two types of manufacturing methods (CNC and 3DP) and two types of CBTs, respectively, and to evaluate their efficacies and accuracies in facilitating ATPI.

## Methods

### Specimen collection

Twenty formalin-preserved cervical vertebrae (range C3–C7) from four human cadavers (three males and one female, ages 45–56) were imaged using a Brilliance CT 64-channel scanner (Philips, Eindhoven, The Netherlands). The in-plane pixel size was 0.5 mm, and the slice thickness was 0.5 mm. All cervical vertebrae involved in this study after the CT scan images showed no significant bone defects which were detected by the same radiologist.

### Preoperative design of CBT

**Fig. 1:**
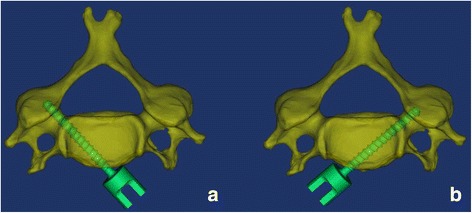
Two types of preoperative CBT designs. **a** CBT0 allows the screw thread to be close to the medial wall of cervical pedicle. **b** CBT0.7 allowed the screw thread to be 0.7 mm lateral from the medial wall of the cervical pedicle

Three-dimensional reconstructions were performed using Mimics 14.11 (Materialise Corp., Leuven, Belgium). There were two CBTs in our study. One CBT allowed the screw (3.5 mm) thread to be close to the medial wall of the pedicle without cortical perforation (CBT0) (Fig. 1a). Since the medial cortical thickness of the cervical pedicle is about 1.4 mm according to the literature [25, 26], in order to allow the screw thread to contact half of the medial cortical wall of the pedicle, a trajectory was designed that allowed the screw thread to be 0.7 mm lateral from the medial wall of the cervical pedicle (CBT0.7) (Fig. 1b).

### Design and manufacturing of PDTs

**Fig. 2:**
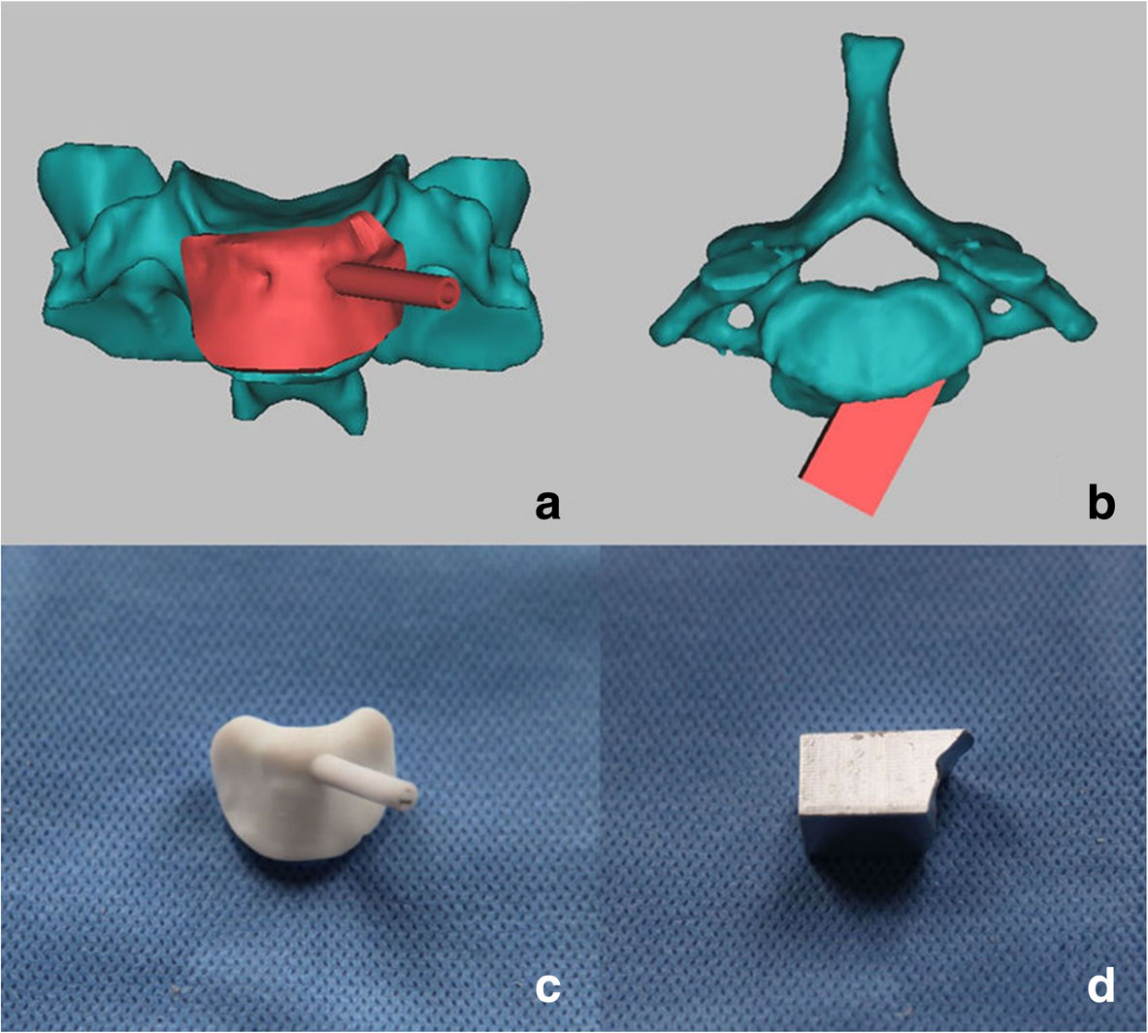
Design and manufacturing of two types of PDTs. The 3DP-produced PDTs were designed and produced with a photosensitive resin (**a**, **c**). CNC-manufactured PDTs were designed and produced with stainless steel (**b**, **d**)

Following the preset ATPI trajectories, PDTs were specifically designed for 3DP or CNC techniques, respectively, with the characteristics of an inverse surface of the anterior vertebral body and a preset guiding tract (Fig. 2a, b) [17]. The 3DP-produced PDTs were manufactured by a stereolithography RP printer RS6000 (Shanghai Union 3D Technology Corp., Shanghai, China) with photosensitive resin (Fig. 2c), and the CNC-manufactured PDTs were machined by a VM650 3 Axis CNC (Bochi Machine Tool Group Corp., Shanxi, China) with stainless steel (Fig. 2d). The manufacturing time and cost of each PDT were both recorded.

### Surgery

**Fig. 3:**
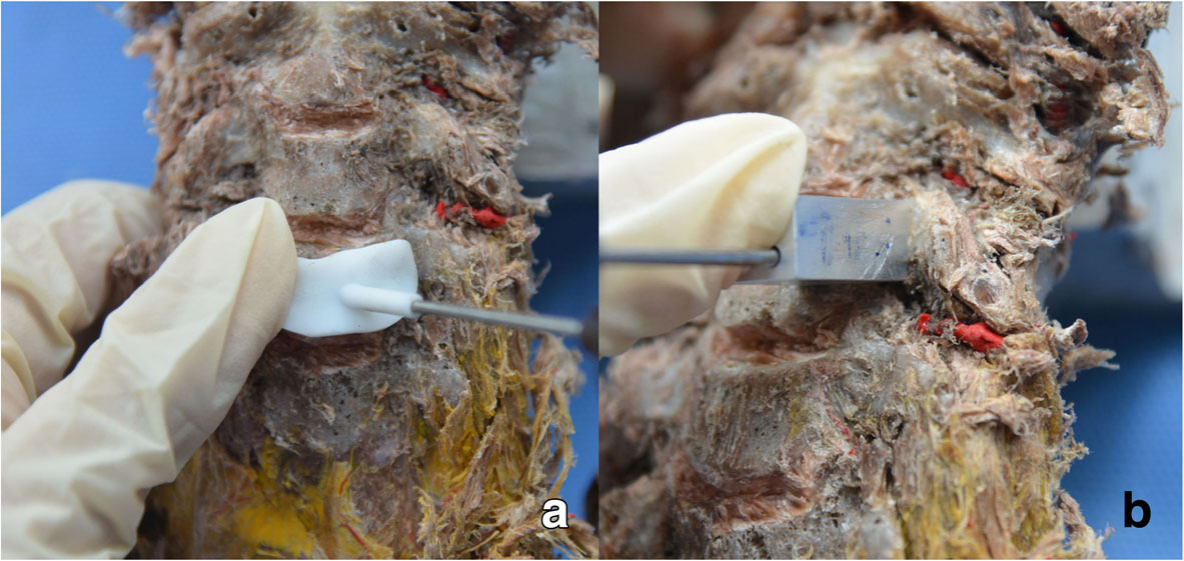
Two-millimeter-diameter K-wires were inserted into cadaveric cervical vertebrae with PDTs. **a** A K-wire was drilled into the cervical pedicle guided by the 3DP-produced PDT. **b** A K-wire was inserted, guided by the CNC-manufactured PDT

After the anterior soft tissue was removed from the vertebrae, a total of 40 pedicle sides were randomly assigned into four groups with two trajectories (CBT0 and CBT0.7) and two manufacturing methods (3DP and CNC) by random numbering chart. The four groups were as follows: group CBT0-3DP, group CBT0-CNC, group CBT0.7-3DP, and group CBT0.7-CNC. Each PDT was compressed slightly to the anterior surface of the cervical vertebrae. A 2-mm-diameter K-wire was subsequently drilled into the cervical pedicle with the assistance of the PDT (Fig. 3). Finally, a 3.5 mm-diameter screw was inserted.

### Assessment of insertion accuracy

Postoperative CT scans were performed for all cadaveric cervical specimens, and the positions of the screws were extracted. The deviations between the preoperatively designed and postoperatively measured screw trajectories were calculated at the entry point and middle point of the pedicle on the transverse and sagittal planes, respectively. The transverse plane’s deviations toward the lateral side were recorded as positive values and the deviations toward the medial side as negative values. The sagittal plane’s deviations toward the superior and inferior sides were recorded as positive and negative values, respectively [16, 17].

A grade was then used to evaluate breaches of the pedicle wall in the sagittal and transverse planes as follows:

- Grade 1: Screw positioned at the center of the pedicle
- Grade 2: Less than one third of the screw cross-section (≤ 1.2 mm with a 3.5-mm-diameter screw) penetrating the cortex
- Grade 3: Between one third and one half of the screw cross-section penetrating the cortex (or deviation < 2 mm)
- Grade 4: More than one half of the screw cross-section penetrating the cortex (or deviation ≥ 2 mm)
- Grade 5: Deviation equal to or greater than the screw diameter [9–11]

Non-critical pedicle breaches corresponded to grades 1 and 2. Critical pedicle breaches, with the potential risk of neurovascular injury, corresponded to grades 3–5 [10].

### Statistical analysis

Results on manufacturing time, price, and deviations are presented as means ± standard deviation. Categorical measurements are presented as numbers. Factorial analysis was used to analyze the absolute deviations of screws between the four groups on the transverse and sagittal planes. The chi-square test was performed to compare the grade of ATPS. SPSS 20 (IBM, the United States) was used for all analyses, and the significance was defined as *p* < 0.05.

## Results

All PDTs were produced successfully by CNC or 3DP. Their manufacture time was 51.65 ± 3.56 min and 109.75 ± 3.88 min, respectively, with significant differences (*t* = 45.35, *p* < 0.001). The cost was 16.44 ± 0.64 dollars and 16.72 ± 1.07 dollars, respectively, with no significant differences (*t* = − 1.796, *p* = 0.082). During the operation, all screws were inserted into the cervical pedicle easily with the assistance of PDTs.

The absolute deviations at the entry point and middle point of the pedicle on the transverse and sagittal planes are presented in Table 1. There were no significant differences between the two types of CBTs on the transverse and sagittal planes at the entry point (*F* = 0.299, *p* = 0.588, and *F* = 0.079, *p* = 0.780, respectively) or at the midpoint of pedicle (*F* = 1.267, *p* = 0.268, and *F* = 0.016, *p* = 0.901, respectively). Similarly, there were also no significant differences between the two manufacturing methods on the transverse and sagittal planes at the entry point (*F* = 0.069, *p* = 0.759, and *F* = 0.025, *p* = 0.875, respectively) or at the midpoint of pedicle (*F* = 1.552, *p* = 0.221, and *F* = 0.601, *p* = 0.443, respectively).

**Table 1:** The absolute deviations at the entry point and middle point of the pedicle on the transverse and sagittal planes (mean ± SD, mm)

Group	Entry point		Midpoint	
	Transverse plane	Sagittal plane	Transverse plane	Sagittal plane
Group CBT0-3DP	0.57 ± 0.28	0.20 ± 0.11	0.82 ± 0.47	0.48 ± 0.45
Group CBT0-CNC	0.44 ± 0.22	0.15 ± 0.10	0.65 ± 0.36	0.40 ± 0.47
Group CBT0.7-3DP	0.52 ± 0.39	0.13 ± 0.12	0.64 ± 0.28	0.49 ± 0.46
Group CBT0.7-CNC	0.54 ± 0.33	0.20 ± 0.13	0.54 ± 0.34	0.61 ± 0.35

**Fig. 4:**
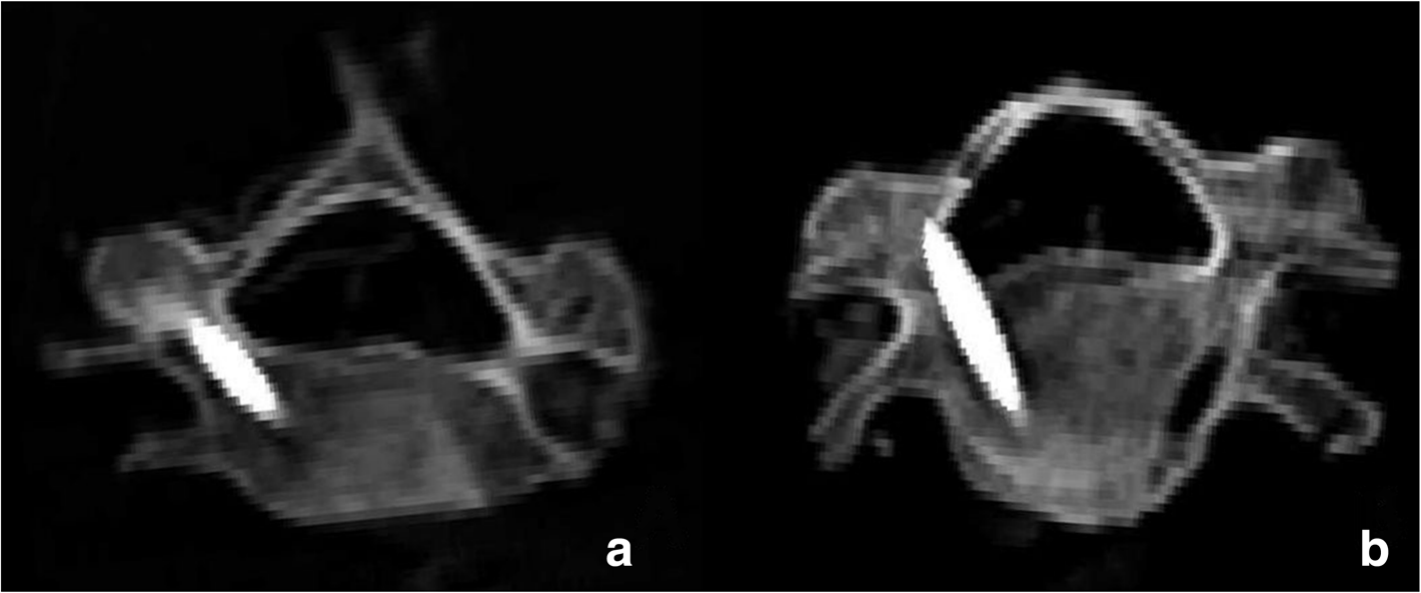
Grades of screw insertion position. **a** Grade 1 indicates a screw positioned at the center of the pedicle without cortical bone breach. **b** Grade 3 indicates a screw that penetrated the cortex between one third and one half of the screw diameter

The grades of screw insertion positions are shown in Table 2. There were nine (90%) in grade 1 and one (10%) in grade 3 in the CBT0-3DP group and eight (80%) in grade 1 and two (20%) in grade 3 in the CBT0-CNC group, whereas all screws were in grade 1 in the CBT0.7-3DP group and the CBT0.7-CNC group (Fig. 4). There were no significant differences between the four groups (*X*^2^ = 7.11, *p* = 0.300).

**Table 2:** Safety of screw insertion classifications

Group	Grade 1	Grade 2	Grade 3	Grade 4	Grade 5
Group CBT0-3DP	9 (90%)	0	1 (10%)	0	0
Group CBT0-CNC	8 (80%)	0	2 (20%)	0	0
Group CBT0.7-3DP	10 (100%)	0	0	0	0
Group CBT0.7-CNC	10 (100%)	0	0	0	0
Total	37	0	3	0	0

## Discussion

In this study, we developed the PDTs with two types of CBTs (CBT0 and CBT0.7) and two manufacturing methods (3DP and CNC). The efficacies and accuracies of these PDTs in facilitating cervical ATPI were further evaluated. These results revealed that the CBT0.7 might be a safer trajectory for cervical ATPI. Meanwhile, with its time-saving and bio-safe merits, CNC might be an alternative manufacturing method for PDTs in cervical ATPI.

In our study, two manufacturing techniques, 3DP and CNC, were used. Actually, the major difference between 3DP and CNC was the additive or subtractive manufacturing techniques they used. To our knowledge, most research related to PDTs used 3DP because of its merits in complex structure formation [15, 27–29]. This method could be more accurate than the free-hand insertion techniques. However, they mainly use nonbiocompatible photosensitive resin. Moreover, they cannot be sterilized at high temperatures. Drilling debris that contacts the wound would bring certain risks. In an attempt to overcome these limitations, Kong et al. [17] used CNC to produce biocompatible metal PDTs and further confirmed their efficacy and accuracy in posterior thoracic pedicle insertion. In our study, CNC was also used to design and produce metal PDTs for cervical ATPI. These CNC-manufactured PDTs were not only biocompatible but also highly precise with free-form surface formations. It should be mentioned that a deep concave could not be created by CNC because of the cutting and routing limitations of the subtractive manufacturing technology. All CNC-manufactured PDT designs should be specially examined and optimized. In our study, since the anterior surface of the cervical vertebrae was relatively flat, there was no deep concave in any of the CNC-manufactured PDTs. The CNC-manufactured PDTs achieved high efficacies and accuracies in facilitating cervical ATPI and were advantageous by saving time in manufacturing compared with 3DP-produced PDTs. However, the total time was about 12 h from acquiring the CT image to obtaining the PDTs, which was a little longer, but might be acceptable for most of the surgeries. Therefore, CNC-manufactured PDTs can be a viable alternative to 3DP-PDTs and could also provide surgeons with a bio-safe, accurate method for cervical ATPI.

In terms of surgical safety, accurate pedicle screw placement and sufficient strength and rigidity of fixation are two major aspects that should concern spinal surgeons. CBT is a novel concept that may provide better implantation strength with the screw’s thread contacting the cortical bone of the pedicle [22–24]. In this circumstance, accurate placement of the pedicle screw with CBT is of vital importance since it has a great probability of pedicle perforation in screw insertion. Therefore, the second objective of this study was to evaluate the accuracy of the two types of CBTs in cervical ATPI.

There were two major considerations about CBT in our study. One was the direction of the CBT. Other than lumbar lateral CBT in the transverse plane [22], CBT in cervical ATPI was designed to be medial because of the lateral structures of the vertebral artery. Previous studies have indicated that lateral perforation was observed more frequently [30, 31], and the consequences of a lateral perforation in the cervical spine, such as cerebral infarction, are serious [32]. In contrast, medial CBT might be safer since the dural sac is located at a distance of approximately 2.4–3.1 mm from the medial pedicle wall [33]. Therefore, we designed the CBT in cervical ATPI to be medial in order to allow the screw thread to approach the medial wall of the pedicle. The second consideration was the setting of the two types of CBTs. One was the CBT0 that allowed the screw thread to be close to the medial wall of the pedicle without cortical perforation. Another was the CBT0.7 that allowed the screw thread to be 0.7 mm lateral from the medial wall of the cervical pedicle. Since the medial cortical thickness of the cervical pedicle is about 1.4 mm [25, 26], CBT0.7 meant that the screw thread contacted half of the medial cortical wall of the pedicle. Moreover, a distance of 0.7 mm lateral from the medial wall of the cervical pedicle would allow the pedicle insertion a deviation tolerance. CBT0.7 would be safe for pedicle insertion because PDT’s insertion accuracy was shown to be between 0.4 and 0.7 mm at the midpoint of the thoracic pedicles in our previous research [17]. Therefore, in our study, we designed two types of CBTs, CBT0 and CBT0.7, and further evaluated their efficacies and accuracies.

Although no significant differences for screw deviations were observed between CBT0 and CBT0.7, three critical breaches (grade 3) occurred with CBT0. The high rate of critical pedicle breach that existed with CBT0 is unacceptable for clinical application, as it may carry a risk of neurovascular injury. Additionally, we found that most screws in grade 1 deviated laterally. This is probably because the medial pedicle wall is thicker. We inferred that when the tip of the K-wire or the screw contacted the medial wall of the cervical pedicle, the thick and tough medial cortical bone would push it outward, which made ATPI with CBT0.7 safe. Therefore, the CBT0.7 was a safer trajectory with less perforation in cervical ATPI.

Our study has several limitations. First, the design of the CBTs was only offset on the transverse plane, and the sagittal plane was not considered. We will address this in future research. Second, the results of the accuracy of the screw insertions with the new trajectories are satisfying, but biomechanical evaluations such as the pullout strength and cyclic fatigue loading were not evaluated in this study, and this needs further research. Third, the PDTs were only performed on cadaveric spines. Clinical studies are needed.

## Conclusion

In summary, CBT0.7 may be a safe and feasible trajectory in cervical ATPI. The bio-safe CNC-PDTs are viable in facilitating cervical ATPI with good feasibility and accuracy.

## Abbreviations

3DP: Three-dimensional printing
ACDF: Anterior cervical decompression and fusion
ATPI: Anterior transpedicular insertion
ATPS: Anterior transpedicular screw
CAN: Computer-assisted navigation
CBT: Cortical bone trajectory
CL: Center line
CNC: Computer numerical control
PDT: Patient-specific drill template
